# Chinese herbal medicine for opioid induced constipation in cancer patients: Protocol for a systematic review

**DOI:** 10.1097/MD.0000000000012594

**Published:** 2018-09-28

**Authors:** Shuo Qi, Hezheng Lai, Yayue Zhang, Qing Dong, Xiaoshu Zhu

**Affiliations:** a Dongzhimen Hospital Affiliate to Beijing University of Chinese Medicine, Beijing, China; b School of Health and Science, Western Sydney University, Campbelltown, New South Wales; c The Chinese Medicine Center, Collaboration Between Beijing University of Chinese Medicine and Western Sydney University.

**Keywords:** cancer, chinese medicine, constipation, opioid

## Abstract

**Background:**

Opioid induced constipation (OIC) is a symptom that is commonly encountered in cancer pain management. Chinese herbal medicine (CHM) has been widely used to improve OIC in China. Many clinical trials indicate that CHM could improve OIC. In this systematic review, we aim to evaluate the effectiveness and safety of CHM for OIC in cancer patients.

**Methods:**

We will search the following electronic databases for randomized controlled trials to evaluate the effectiveness and safety of CHM for OIC in cancer patients: CENTRAL, EMBASE, MEDILINE, CINAHL and China National Knowledge Infrastructure. Each database will be searched from inception to June 2018. The entire process will include study selection, data extraction, risk of bias assessment and meta-analyses.

**Results:**

This proposed study will evaluate the effectiveness and safety of CHM for OIC. The outcomes will include change in bowel movements, quality of life and adverse events.

**Conclusions:**

This proposed systematic review will evaluate the existing evidence on the effectiveness and safety of CHM for OIC in cancer patients.

**Dissemination and ethics:**

The results of this review will be disseminated through peer-reviewed publication. Because all of the data used in this systematic review and meta-analysis has been published, this review does not require ethical approval. Furthermore, all data will be analyzed anonymously during the review process.

## Abstract 2

Supplemental Digital Content is available in the text

## 1 Introduction

Pain is one of the most common and dreaded cancer related symptoms.^[1]^ A meta-analysis found that pain was reported in 59% of patients undergoing cancer treatment, and that it was reported with greater frequency by patients in advanced stages of cancer.^[2]^ Unrelieved pain severely affects quality of life in cancer patients.^[3]^ Opioids such as morphine, oxycodone, and fentanyl, are recommended by the World Health Organization (WHO) and are indicated in the National Comprehensive Cancer Network Guidelines (NCCN Guidelines) for Adult Cancer Pain.

However, opioid use is often associated with adverse events.^[4]^ Constipation is a symptom of opioid induced bowel dysfunction that patients often find intolerable yet is often anticipated with opioid treatment for cancer related pain. Persistent constipation seriously affects the quality of life (QOL) of cancer patients and may lead to anxiety and nervousness.^[5–7]^ Therefore, the management of opioid induced constipation (OIC) is of critical importance.

Use of laxatives, stool softeners, and lifestyle management, including the introduction of a fiber-rich diet, increase in water intake, and physical exercise are recommended for patients with OIC.^[8]^ Despite these existing management methods, more than 80% of cancer patients prescribed with opioids still suffer from constipation.^[9,10]^ Opioid antagonists have been developed and are currently used clinically to improve OIC.^[11]^ However, a recent systematic review found that use of opioid antagonists increases the risk of adverse events.^[11]^

Chinese Medicine is a complementary and alternative therapy that has been used in integration with Western biomedicine for the management of cancer and its symptoms. Many kinds of Chinese Medicine therapies are employed for treating OIC, including orally administered Chinese herbal medicine (CHM), topical use CHM, acupuncture, and moxibustion. The results of clinical trials of CHM for the treatment of OIC have been inconsistent. These inconclusive results may be due to inconsistencies in these studies’ methodologies. This review aims to systematically review all randomized controlled trials (RCTs) to assess the effectiveness and safety of orally administered CHM for OIC in cancer patients.

## 2 Materials and methods

This systematic review protocol has been registered on PROSPERO as CRD42018106706. The Protocol follows the Cochrane Handbook for Systematic Reviews of Interventions and the Preferred Reporting Items for Systematic Reviews and Meta-Analysis Protocol (PRISMA-P) statement guidelines.^[12]^ We will describe the changes in our full review if needed.

## 3 Inclusion criteria for study selection

### 3.1 Type of studies

This review will include clinical RCTs of CHM therapy for OIC in cancer patients without any language or publication status restrictions. Non-RCTs, quasi-RCTs, case series, case reports, crossover studies, uncontrolled trials, and laboratory studies will not be included.

### 3.2 Type of participants

Cancer patients, male or female, of any age with a diagnosis of OIC will be included. Pregnant and lactating women will be excluded. Patients with intestinal stenosis caused by rectum or colon organic diseases (such as cancer, Crohn's disease, colon polyps, intestinal tuberculosis, and so on) will be excluded. Patients with acute intestinal obstruction will be excluded.

### 3.3 Type of interventions

Interventions will include any type of orally administered CHM. Studies of CHM combined with other treatments such as acupuncture will be considered for exclusion.

Control: placebo, no intervention and orally administered western biomedicine. Studies of control intervention such as enema will be considered for exclusion.

### 3.4 Type of outcome measures

#### 3.4.1 Primary outcomes

The primary outcome measure will be change in bowel movements.

#### 3.4.2 Secondary outcome

The secondary outcome measures will include quality of life, number, and type of adverse events.

## 4 Search methods for the identification of studies

### 4.1 Electronic searches

We will search the following databases for relevant trails:

- Cochrane Central Register of Controlled Trials (CENTRAL);
- CINAHL (Cumulative Index of Nursing and Allied Health Literature, from 1937 to present);
- EMBASE (Excerpta Medica database, from 1947–present);
- Ovid MEDLINE ALL (Ovid Medical Literature Analysis and Retrieval System Online, from 1946–present);
- CNKI (China National Knowledge Infrastructure Database, from 1979–present).

### 4.2 Data collection and analysis

#### 4.2.1 Study identification

We will use EndNote X8 software to manage the records of searched electronic databases. The initial selection will involve scanning of the titles and abstracts of the retrieved studies. The full text of relevant studies will then be reviewed for study inclusion, in accordance with the inclusion criteria, by 2 authors (SQ and QD). Potentially relevant articles will be reviewed independently by 2 authors (SQ and XZ) to determine if they meet the prespecified criteria. Any disagreement between authors will be resolved by consensus with a third author. The study selection procedure will follow and be recorded in the PRISMA flow chart. All the evidence will be assessed by The Grading of Recommendations Assessment, Development and Evaluation (GRADE).

#### 4.2.2 Data extraction and management

According to the inclusion criteria, a standard data collection form will be made before data extraction. The following data will be extracted by 2 authors (SQ and QD):

- General information: research identification, publication year, the title of the study, first author;
- Study methods: study design, sample size, randomization method, allocation concealment, blinding, incomplete report or selecting report, other sources of bias;
- Participants: inclusion and exclusion criteria;
- Intervention: CHM name and dosage, treatment details, treatment duration, and frequency;
- Control: type of control drugs, treatment details, treatment duration, and frequency;
- Outcomes: primary, secondary, and safety outcomes.

#### 4.2.3 Risk of bias assessment

The risk of bias in included studies will be assessed independently by 2 reviewers (SQ and XZ). The following domains will be assessed: random sequence generation, allocation concealment, blinding of participants, blinding of outcome assessment, incomplete outcome data, selective outcome reporting, and other bias.

#### 4.2.4 Data analysis

We will use Review Manager Software (Review Manager (RevMan) [Computer program]. Version 5.3. Copenhagen: The Nordic Cochrane Centre, The Cochrane Collaboration, 2014) provided by Cochrane Collaboration for data synthesis and analysis by 2 authors (SQ and QD). We will summarize data using risk radio (RR) with 95% confidence intervals (CI) for binary outcomes. If different measurement scales are used, standardized mean difference analyses will be performed. We will consider heterogeneity to be substantial if the *I*^*2*^ statistic is greater than 50%. Data from individual trials will be combined for meta-analysis if the interventions, patient groups and outcomes are sufficiently similar. Meta-analysis will not be performed if the *I*^*2*^ statistic is ≥85%. We will use a random-effects model for meta-analysis unless the degree of heterogeneity is readily explainable. If the *I*^*2*^ statistic is less than 25% a fixed-effect model will be used.

Sensitivity analyses: heterogeneity may be due to the presence of 1 or more outlier studies with results that conflict with the rest of the studies. We will perform sensitivity analyses excluding outlier studies. In addition, we plan to perform sensitivity analysis to explore the influence of trial quality on effect estimates. The quality components of methodology include adequacy of generation of allocation sequence, concealment of allocation, and the use of intention-to-treat analysis.

Subgroup analyses: if data permits, we will perform the following subgroup analyses.

- Different types of outcome measures;
- Different types of control therapies;
- Treatment duration.

#### 4.2.5 Publication bias

If sufficient number of trials (more than 10 trials) are found, we will generate funnel plots (effect size against standard error) to investigate publication bias.

#### 4.2.6 Ethics and dissemination

The data used in this systematic review will be collected from published studies. Based on this, the study does not require ethical approval.

## Acknowledgment

The authors would like to thank all the researcher in our working group.

Appendix A. CENTRAL Search Strategy

## Author contributions

SQ, XZ contributed on methodology and are the guarantors of the review.

SQ, HL, XZ, YZ, and QD contributed on data search, analysis, and statistics.

HL contributed on the language editing.

**Methodology:** Shuo Qi, Xiaoshu Zhu.

**Resources:** Yayue Zhang, Qing Dong.

**Writing – original draft:** Shuo Qi.

**Writing – review & editing:** Hezheng Lai, Xiaoshu Zhu.

Shuo Qi orcid: 0000-0001-7559-4606

## Supplementary Material

Supplemental Digital Content

## References

[1] Sheinfeld Gorin S Krebs P Badr H Meta-analysis of psychosocial interventions to reduce pain in patients with cancer. J Clin Oncol 2012;30:539–47. 22253460 10.1200/JCO.2011.37.0437 PMC6815997

[2] van den Beuken-van Everdingen MH de Rijke JM Kessels AG Prevalence of pain in patients with cancer: a systematic review of the past 40 years. Ann Oncol 2007;18:1437–49. 17355955 10.1093/annonc/mdm056

[3] Mantyh PW Cancer pain and its impact on diagnosis, survival and quality of life. Nat Rev Neurosci 2006;7:797–809. 16988655 10.1038/nrn1914

[4] McNicol E Horowicz-Mehler N Fisk RA Management of opioid side effects in cancer-related and chronic noncancer pain: a systematic review. J Pain V 4 2003;231–56. 10.1016/s1526-5900(03)00556-x 14622694

[5] Te Boveldt N Vernooij-Dassen M Burger N Pain and its interference with daily activities in medical oncology outpatients. Pain Physician 2013;16:379–89. 23877454

[6] Paice JA Ferrell B The management of cancer pain. CA Cancer J Clin 2011;61:157–82. 21543825 10.3322/caac.20112

[7] Pachman DR Barton DL Swetz KM Troublesome symptoms in cancer survivors: fatigue, insomnia, neuropathy, and pain. J Clin Oncol 2012;30:3687–96. 23008320 10.1200/JCO.2012.41.7238

[8] National Collaborating Center for C. National Institute for Health and Clinical Excellence: Guidance. Opioids in Palliative Care: Safe and Effective Prescribing of Strong Opioids for Pain in Palliative Care of Adults. Cardiff (UK): National Collaborating Center for Cancer (UK) Copyright 2012, (c) National Collaborating Centre for Cancer; 2012 https://www.ncbi.nlm.nih.gov/pubmed/23285502.

[9] Coyne KS LoCasale RJ Datto CJ Opioid-induced constipation in patients with chronic noncancer pain in the USA, Canada, Germany, and the UK: descriptive analysis of baseline patient-reported outcomes and retrospective chart review. ClinicoEconomics and outcomes research: CEOR 2014;6:269–81. 24904217 10.2147/CEOR.S61602 PMC4041290

[10] Diego L Atayee R Helmons P Novel opioid antagonists for opioid-induced bowel dysfunction. Expert Opin Investig Drugs 2011;20:1047–56. 10.1517/13543784.2011.592830 21663526

[11] Candy B Jones L Vickerstaff V Mu-opioid antagonists for opioid-induced bowel dysfunction in people with cancer and people receiving palliative care. Cochrane Database Syst Rev 2018;6:Cd006332. 29869799 10.1002/14651858.CD006332.pub3 PMC6513061

[12] Shamseer L Moher D Clarke M PRISMA-P Group. Preferred reporting items for systematic review and meta-analysis protocols (PRISMA-P) 2015: elaboration and explanation. BMJ 2015;349:g7647. 10.1136/bmj.g7647 25555855

