# Supplementary material for: Chinese herbal medicine for opioid induced constipation in cancer patients: Protocol for a systematic review
Source: Medicine (Baltimore). 2018 Sep 28;97(39):e12594. doi: 10.1097/MD.0000000000012594 (PMC6181624; doi:10.1097/MD.0000000000012594)
Supplement: Supplemental Digital Content [file medi-97-e12594-s001.docx]

**Appendix A. CENTRAL Search Strategy**

#1 MeSH descriptor: [Medicine, Chinese Traditional] explode all trees

#2 MeSH descriptor: [Drugs, Chinese Herbal] explode all trees

#3 TCM or CM or CHM or Chinese herbal medicine or herbal medicine or Chinese medicine or traditional Chinese medicine

#4 #1 or #2 or #3

#5 cancer or tumor or tumour or neoplasm or malignant neoplasm

#6 MeSH descriptor: [Neoplasms] explode all trees

#7 #5 or #6

#8 opioid or oxycodone or oxycontin or morphine or ms contin or fentanyl or sufentanil or tramadol

#9 MeSH descriptor: [Analgesics, Opioid] explode all trees

#10 MeSH descriptor: [Opioid-Related Disorders] explode all trees

#11 MeSH descriptor: [Tramadol] explode all trees

#12 MeSH descriptor: [Oxycodone] explode all trees

#13 MeSH descriptor: [Fentanyl] explode all trees

#14 MeSH descriptor: [Sufentanil] explode all trees

#15 MeSH descriptor: [Morphine] explode all trees

#16 #8 or #9 or #10 or #11 or #12 or #13 or #14 or #15

#17 MeSH descriptor: [Constipation] explode all trees

#18 constipation or OIC

#19 #17 or #18

#20 #4 and #7 and #16 and #19

**Abbreviations:** TCM = Traditional Chinese Medicine, CM = Chinese Medicine, CHM = Chinese Herbal Medicine, OIC = Opioid Induced Constipation.
